# Tubeimoside I Ameliorates Myocardial Ischemia-Reperfusion Injury through SIRT3-Dependent Regulation of Oxidative Stress and Apoptosis

**DOI:** 10.1155/2021/5577019

**Published:** 2021-11-09

**Authors:** Dingyi Lv, Minghao Luo, Zhe Cheng, Ruiyu Wang, Xiyang Yang, Yongzheng Guo, Longxiang Huang, Xiang Li, Bi Huang, Jian Shen, Suxin Luo, Jianghong Yan

**Affiliations:** ^1^Department of Vascular Cardiology, The First Affiliated Hospital of Chongqing Medical University, Chongqing 400010, China; ^2^Institute of Life Sciences, Chongqing Medical University, Chongqing 400010, China

## Abstract

Myocardial ischemia-reperfusion injury (MIRI) is a phenomenon that reperfusion leads to irreversible damage to the myocardium and increases mortality in acute myocardial infarction (AMI) patients. There is no effective drug to treat MIRI. Tubeimoside I (TBM) is a triterpenoid saponin purified from Chinese traditional medicine tubeimu. In this study, 4 mg/kg TBM was given to mice intraperitoneally at 15 min after ischemia. And TBM treatment improved postischemic cardiac function, decreased infarct size, diminished lactate dehydrogenase release, ameliorated oxidative stress, and reduced apoptotic index. Notably, ischemia-reperfusion induced a significant decrease in cardiac SIRT3 expression and activity, while TBM treatment upregulated SIRT3's expression and activity. However, the cardioprotective effects of TBM were largely abolished by a SIRT3 inhibitor 3-(1H-1,2,3-triazol-4-yl) pyridine (3-TYP). This suggests that SIRT3 plays an essential role in TBM's cardioprotective effects. *In vitro*, TBM also protected H9c2 cells against simulated ischemia/reperfusion (SIR) injury by attenuating oxidative stress and apoptosis, and siSIRT3 diminished its protective effects. Taken together, our results demonstrate for the first time that TBM protects against MIRI through SIRT3-dependent regulation of oxidative stress and apoptosis. TBM might be a potential drug candidate for MIRI treatment.

## 1. Introduction

Acute myocardial infarction (AMI) induced by coronary artery occlusion remains a leading cause of morbidity and mortality worldwide [1]. And it brings serious psychological and economic burden to patients who suffer from the condition. Early reperfusion therapy, such as thrombolysis and percutaneous coronary intervention (PCI), has become a routine treatment strategy for myocardial infarction [2]. However, reperfusion may lead to irreversible damage to the myocardium and increases mortality in AMI patients. This phenomenon is called myocardial ischemia-reperfusion injury (MIRI) [3]. The underlying mechanisms of MIRI are complex. The main involved mechanisms include overproduction of reactive oxygen species (ROS), calcium overload, inflammatory reactions, mitochondrial dysfunction, and activation of apoptosis [4–8]. Moreover, more and more evidences show that ROS is the major pathological process leading to MIRI [4, 9]. ROS are produced mainly by mitochondria, and excessive ROS leads to apoptosis of cardiomyocytes [8, 10]. Therefore, it is a vital preventional treatment of MIRI to reduce the apoptosis induced by oxidative stress during reperfusion.

Sirtuins are a family of deacetylase consisting of seven members (SIRT1-7), and their activities are dependent on nicotinamide adenine dinucleotide (NAD^+^). SIRT3 primarily localizes in the mitochondria, and it possesses the most robust deacetylase activity among all the mitochondrial sirtuins [11–13]. SIRT3 mediates most mitochondrial proteins' deacetylation [14]. SIRT3 is highly expressed in the heart and plays vital roles in MIRI [15, 16]. In artery occlusion-mediated MI, SIRT3's expression is inhibited [17]. SIRT3^−/−^ mice are more susceptible to MIRI, and ischemia-reperfusion brings larger infarct size in SIRT3^−/−^ mice [18]. In MIRI, ROS opens mitochondrial permeability transition pore (mPTP), makes mitochondrial swelling, activates apoptotic pathway, and ultimately leads to cell death [19]. SIRT3 targets and deacetylates cyclophilin D which is a regulatory subunit of mPTP; hence, it delays mPTP's opening and alleviates MIRI [20, 21]. In addition, SIRT3 reduces MIRI by increasing activities of antioxidant enzymes such as SOD2 [22, 23]. SIRT3 also inhibits apoptosis by blocking translocation of Bax [24]. Taking together, all these results suggest that SIRT3 exerts vital protective functions in MIRI.

Tubeimoside I (TBM) is a triterpenoid saponin purified from tubeimu (tuber of *Bolbostemma paniculatum* (Maxim.) Franquet) [25, 26]. In traditional Chinese medicine, tubeimu has been used for over 1000 years. It worked well in treating inflammatory diseases, snake bites, acute mastitis, tumors, and detoxification [27–31]. At present, there are 63 articles related with TBM. And most of these articles are about TBM's antitumor activity. Generally, TBM induces apoptosis and cell cycle arrest in kinds of cancer cells [27]. The signaling pathways involved in TBM's antitumor activity are ROS/cytochrome C/Caspase-3, ROS/MAPK, MAPK-JNK, MAPK/p38, PI3K/Akt, AKT/mTOR, p53/MDM2, NF-*κ*B, VEGFA/VEGFR-2/ERK, MEK/ERK, Wnt/*β*-catenin, CXCL12-CXCR4, and Akt–mTOR–eEF-2K [27]. However, its effect on MIRI is unclear. This study is aimed at investigating whether TBM ameliorates MIRI and evaluating the role of SIRT3 in this process both *in vivo* and *in vitro*.

In the present study, we observed that TBM alleviated IR-induced oxidative stress and apoptosis both *in vivo* and *in vitro*. And its protective effects were abolished by SIRT3 inhibition. These results demonstrated that TBM ameliorated MIRI through SIRT3-dependent regulation of oxidative stress and apoptosis, indicating TBM is a promising drug to treat MIRI.

## 2. Materials and Methods

### 2.1. Materials

TBM (BP1415) was bought from Chengdu Biopurify Technology Development Co. LTD. (http://www.biopurify.cn/). 3-TYP (HY-108331) was bought from MedChemExpress (Monmouth Junction, NJ, USA). Primary antibodies against SIRT3 (10099-1-AP), SOD2 (24127-1-AP), Bcl-2 (12789-1-AP), GAPDH (60004-1-Ig), Bax (50599-2-Ig), NAD(P)H:quinone oxidoreductase 1 (NQO1, 11451-1-AP), COX-IV (11242-1-AP), and nuclear factor erythroid 2-related factor 2 (Nrf2, 16396-1-AP) and secondary antibodies (goat anti-rabbit, SA00001-2; goat anti-mouse, SA00001-1) were bought from Proteintech Group (Chicago, IL, USA). Cytochrome C (#4280) and Cleaved Caspase-3 (#9664) primary antibodies were bought from CST (Danvers, MA, USA). NADPH oxidase 2 (NOX2, sc-130543) primary antibodies were bought from Santa Cruz Biotechnology (Santa Cruz, CA, USA). Ac-SOD2 (ab137037) primary antibodies were bought from Abcam (Cambridge Biomedical Campus, Cambridge, UK). TUNEL Assay Kit (C1086), Cell Mitochondria Isolation Kit (C3601), 4′,6-diamidino-2-phenylindole (DAPI) solution (C1005), Tissue Mitochondria Isolation Kit (C3606), and Reactive Oxygen Species Assay Kit (S0033S) were purchased from Beyotime (Shanghai, China). Lactate dehydrogenase (LDH) assay kit (A020-2-2), malondialdehyde (MDA) assay kit (A003-1-2), and superoxide dismutase (SOD) assay kit (A001-3-1) were purchased from Nanjing Jiancheng Bioengineering Institute (Nanjing, China). CCK8 (Cell Counting Kit-8) (B34304) were bought from Bimake (Houston, TX, USA). 2,3,5-Triphenyltetrazolium chloride (TTC) (G3005) was purchased from Solarbio (Beijing, China). Other reagents were purchased from common reagent manufacturers. All used reagents are of analytical purity.

### 2.2. Animal Experiments

Wild-type male C57BL/6 mice were used in this study. The laboratory animal center of Chongqing Medical University supplied mice. Mice were kept under standard specific pathogen-free conditions and were allowed free access to water and chow. Animal experiments were approved by the Animal Ethic Committee of Chongqing Medical University. Mice were randomly divided. The following groups were set up: (1) sham group: mice underwent the sham operation (all operations were the same with those in the IR group except ligation) and were treated with vehicle (5% saline); (2) TBM group: TBM (4 mg/kg) was given via intraperitoneal injection; (3) IR group: mice underwent IR operation and treated with vehicle (5% saline); (4) IR+TBM group: mice underwent IR operation and treated with 4 mg/kg TBM via intraperitoneal injection at 15 min after IR operation; and (5) IR+TBM+3-TYP group: mice were pretreated with 3-TYP (a dose of 50 mg/kg every 2 days for a total of three doses was intraperitoneally injected prior to the IR surgery), treated with TBM (4 mg/kg via intraperitoneal injection at 15 min after IR operation) and then surgery was performed as previously described [32]. Briefly, mice were anesthetized with 2–3% isoflurane. Animals were intubated orally and connected to a mouse miniventilator (MiniVent 845, Harvard Apparatus, Holliston, MA, USA) with PE-90 tubing. To keep the mice under anesthesia, 1% isoflurane was continuously provided. Core body temperature was maintained at 37°C using a thermo heating pad and monitored with a rectal thermometer. A median sternotomy was performed, and the left anterior descending coronary artery (LAD) was visualized. An 8.0 nylon suture (Prolene; Ethicon, Norderstedt, Germany) was placed around the vessel and a loose loop was formed to induce ischemia. For easier ligature release and better reperfusion, a PE-10 tube was placed on the LAD before loop was tied. Ligation was visually confirmed by appearance of pale and bulging myocardium in the area at risk. The PE-10 tube was removed after 30 minutes to allow reperfusion of the myocardium which was indicated by a bright red color within the LAD. Three hours after reperfusion, supernatant plasma and the hearts were harvested and stored at −80°C for subsequent tests. For cardiac function, apoptotic index, and infarct size determination, all sutures and tubes were taken out and incisions were sutured after ischemia, and then, mice were set free and reperfusion lasted for 24 h. Details of *in vivo* experimental protocol are shown in Figure S1.

### 2.3. Cell Culture and Treatment

H9c2 cells were cultivated as described previously [33]. Simulated ischemia-reperfusion (SIR) was performed using ischemic DMEM (DMEM without glucose and serum and dissolved oxygen in the culture media was expelled by filling nitrogen) and low-oxygen incubator as described previously [33–37]. Briefly, H9c2 cells were inoculated and cultivated for 24 h, starved for 4 h, and pretreated with TBM for 1 h. Then, cells were treated in ischemic DMEM and incubated in low-oxygen incubator (Thermo Scientific, Waltham, MA, USA) for 2 h (air conditions are 5% CO_2_, 1% O_2_, and 94% N_2_) and reperfusion was initiated by changing ischemic DMEM into complete DMEM (containing 4.5 g glucose and 10% FBS) and incubated using 95% air+5% CO_2_. Cells were harvested at 4 h (for oxidative stress measurement and western blotting) or 24 h (for cell viability and apoptotic index detection) after reperfusion. Figure S2 shows the detailed *in vitro* experimental protocol.

### 2.4. Echocardiography

Echocardiography was conducted to assess the cardiac function at 24 h after reperfusion as described before [33]. Briefly, mice were anaesthetized and laid on handling platform warmed to 37°C. Hair removing cream was used to remove the chest fur. Ultrasound probe (L8-18i-D PROBE, GE Healthcare, Boston, MA, USA) was used to image heart long axis. Then, EDV (end diastolic volume), ESV (end systolic volume), LVIDs (left ventricle internal diameter at systole), and LVIDd (left ventricle internal diameter at diastole) were measured with M-mode images. The LVEF (left ventricle ejection fraction) and LVFS (left ventricle fractional shortening) were calculated according to instrument instructions. An investigator blinded to the treatment performed these measurements.

### 2.5. TTC Staining

Myocardial infarct size was measured by TTC staining. Briefly, after 30 min of ischemia and 24 h of reperfusion, mouse hearts were excised and frozen at -20°C for 15 min. Then, hearts were cut into 1 mm thick transverse slices, which were incubated in 2% TTC (Solarbio, Beijing, China) at 37°C for 10 min. Images were photographed using a digital camera. The areas of infarcted myocardium (white region) and viable tissue (red region) were evaluated using Image Pro Plus (Media Cybernetics). The degree of infarct was expressed as a percentage of infarcted myocardium/the LV area.

### 2.6. MDA, SOD, and LDH Measurements

To determine systemic oxidative stress, MDA, SOD, and LDH measurements were carried using commercial kits. The Multiskan Spectrum Microplate Spectrophotometer (Thermo Scientific, Waltham, MA, USA) was used to read spectrophotometrical values. MDA, SOD, and LDH concentrations were calculated following manufacturer's instructions.

### 2.7. Western Blotting

Samples were lysed in ice-cold lysis buffer for 30 min and centrifugated at 12,000 × *g* for 15 min. For mitochondrial protein extraction, commercial kits were used. Mitochondria/Cytosol Fractionation Kits (C3601 for cells, C3606 for tissues) were bought from Beyotime (Shanghai, China), and mitochondrial or cytoplasmic proteins were extracted according to the manufacturer's instruction. Protein concentration was determined using the Bradford method. Finally, target proteins were determined with western blotting. Detailed western blotting protocol can be referred from previous literatures [33, 38, 39].

### 2.8. Terminal Deoxynucleotidyl Transferase dUTP Nick End Labeling (TUNEL) Assay

TUNEL assay was used to determine apoptotic cardiomyocytes. Briefly, 4% paraformaldehyde-fixed myocardial tissues were embedded with paraffin and sectioned into 5 *μ*m thick sections. Sections were put onto slides and deparaffinized with xylene. To increase permeability, sections were treated with proteinase K solution (20 *μ*g/ml, 37°C, 30 min) and 0.5% Triton X-100 (room temperature, 5 min). Then, DNA breaks were labeled in TUNEL reaction mixture (37°C, 60 min, light-free). After being rinsed with PBS for 3 times, slides were washed with PBS and the nuclei were labeled with DAPI. Fluorescence microscopy was used to image sections. Apoptotic index was calculated as the ratio of green fluorescence cells/blue fluorescence cells. For each specimen, at least 10 different random fields were selected to do analysis.

### 2.9. CCK8 Assay

CCK8 assay was used to evaluate cell viability. Briefly, a density of 1 × 10^4^ cells/well H9c2 were inoculated and cultured in 96-well plates for 24 h. Then, cells were treated as described in Cell Culture and Treatment. For CCK8 assay, 100 *μ*l mixture (90 *μ*l DMEM+10 *μ*l CCK8 solution) was added to each well. The mixture was incubated at 37°C for a period of time (1-4 h). Then, a microplate spectrophotometer (Thermo Scientific, Waltham, MA, USA) was used to read OD_450_ values. TBM's effects on cell viability were expressed as the percentage of OD_450_ values compared with the control group, which was set at 100%.

### 2.10. ROS Staining

To check TBM's effect on oxidative stress directly, ROS staining was used to detect intracellular ROS in H9c2 cells. Briefly, a density of 1 × 10^4^ cells/well H9c2 was seeded in 96-well plates and cultured for 24 h. After treatment, 2,7-dichlorodi-hydrofluorescein diacetate (DCFH-DA) solution was added to each well. Then, cells were incubated at 37°C for 20 min (light-free). Fluorescence microscope (Leica, Heidelberg, Germany) was used to image cells washed with PBS. The excitation is 488 nm, and the emission is 525 nm. For each specimen, at least 10 different random fields were imaged for analysis. Image Pro Plus (Media Cybernetics) was used to determine the relative fluorescence intensity of ROS per field.

### 2.11. Transient Transfection

BLOCK-iT™ RNAi Designer (Thermo Scientific, Waltham, MA, USA) was used to design three siRNAs direct against SIRT3, and siRNAs were synthesized by RiboBio (Guangzhou, China). Their target sequences were shown as follows: CAGCAAGGTTCTTACTACA (siSIRT3-1), CTGAATCGGTACAGAAATC (siSIRT3-2), and GCAACCTTCAGCAGTATGA (siSIRT3-3). Lipofectamine RNAiMax (Thermo Scientific, Waltham, MA, USA) was used to transfect siRNAs into cells. Thirty-six hours after transfection, transfected cells were treated by SIR as described in Cell Culture and Treatment.

### 2.12. Statistical Analysis

All numerical data are expressed as means ± standard deviation (SD). GraphPad Prism 8.0 software was used to perform all analyses. Student's *t*-test was used to evaluate differences between two groups, and one-way analysis of variance (ANOVA) followed by Dunnett's post hoc test was used to evaluate differences among multiple groups. *p* < 0.05 was considered as statistically significant.

## 3. Results

### 3.1. TBM Improves Cardiac Function and Reduces Infarct Size and Plasma LDH Level in IR Mice

**Figure 1:**
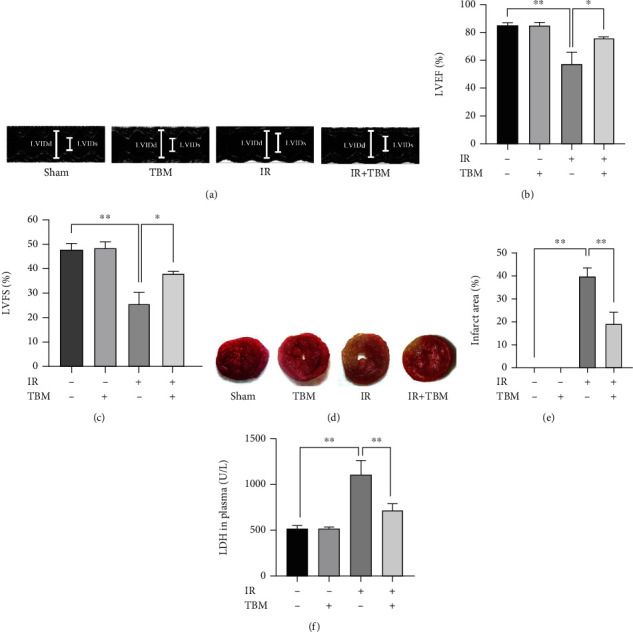
TBM ameliorated cardiac dysfunction and reduced infarct size and plasma LDH level in IR mice. TBM (4 mg/kg) was given at 15 min after ischemia via intraperitoneal injection. At 24 h after IR, cardiac function, infarct size, and plasma LDH level were examined as described. (a) Representative images of M-mode echocardiography. (b) Statistical analysis of LVEF. (c) Statistical analysis of LVFS. (d) Representative images of cardiac sections stained by TTC. Noninfarcted region is red, and the infarcted region is white. (e) Statistical analysis of myocardial infarct size. Infarct area (%) = infarcted region/LV area∗100%. (f) Statistical analysis of plasma LDH level. All numerical data are expressed as means ± SD, *n* = 6. ^∗^*p* < 0.05; ^∗∗^*p* < 0.01.

As Figures 1(a)–1(c) show, IR induced a significant reduction in LVEF and LVFS compared with the sham group, while TBM improved cardiac function by increasing LVEF and LVFS. To detect TBM's effects on the heart more directly, TTC staining was used to detect myocardial infarction size and plasma LDH levels were also measured. As shown in Figures 1(d)–1(f), infarct size and plasma LDH levels increased by IR were significantly reduced by TBM.

### 3.2. TBM Reduces IR-Caused Cardiac Oxidative Stress *In Vivo*

**Figure 2:**
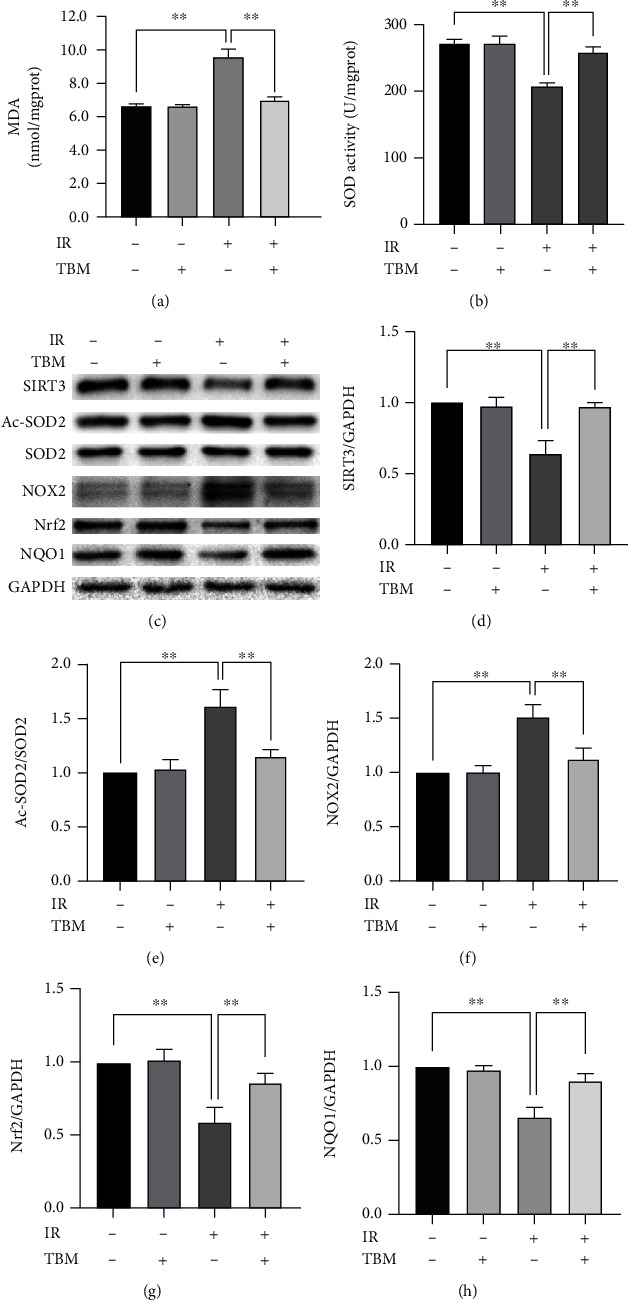
TBM reduced IR-caused cardiac oxidative stress *in vivo*. TBM (4 mg/kg) was given at 15 min after ischemia via intraperitoneal injection. At 3 h postreperfusion, MDA content and SOD activity in ischemic myocardial tissue were measured using related kits. Target proteins were examined by western blotting. (a) Statistical analysis of MDA. (b) Statistical analysis of SOD activity. (c) Representative western blotting results. (d) SIRT3/GAPDH's statistical analysis. (e) Ac-SOD2/SOD2's statistical analysis. (f) NOX2/GAPDH's statistical analysis. (g) Nrf2/GAPDH's statistical analysis. (h) NQO1/GAPDH's statistical analysis. All numerical data are expressed as means ± SD, for MDA and SOD activity, *n* = 6; for western blotting, *n* = 4. ^∗∗^*p* < 0.01.

As Figure 2(a) shows, MDA content in cardiac tissue was increased by IR, indicating that IR induced oxidative stress in the heart. IR-induced oxidative stress was significantly inhibited by TBM (Figure 2(a)). Generally, oxidative stress is caused by the imbalance between ROS production and scavenging ability. To figure out TBM's effects on oxidative stress, we detected TBM's effects on both ROS production and ROS scavengers. On the one hand, TBM decreased ROS production by inhibiting NOX2 which is an essential superoxide producer and contributes to oxidative stress under various pathological conditions (Figures 2(c) and 2(f)). On the other hand, TBM increased SOD activity (Figure 2(b)) and enhanced expression of antioxidant factors Nrf2 and NQO1 (Figures 2(c), 2(g), and 2(h)). As SIRT3 can elevate activity of SOD2 by deacetylation, we also detected SIRT3 and Ac-SOD2 by western blotting. As Figures 2(c)–2(e) showed, IR-inhibited SIRT3 and IR-enhanced Ac-SOD2/SOD2 were all reversed by TBM treatment. These results indicated TBM reduced IR-caused cardiac oxidative stress *in vivo*.

### 3.3. TBM Reduces IR-Caused Cardiac Apoptosis and Inhibits IR-Activated Apoptotic Signaling Pathway

**Figure 3:**
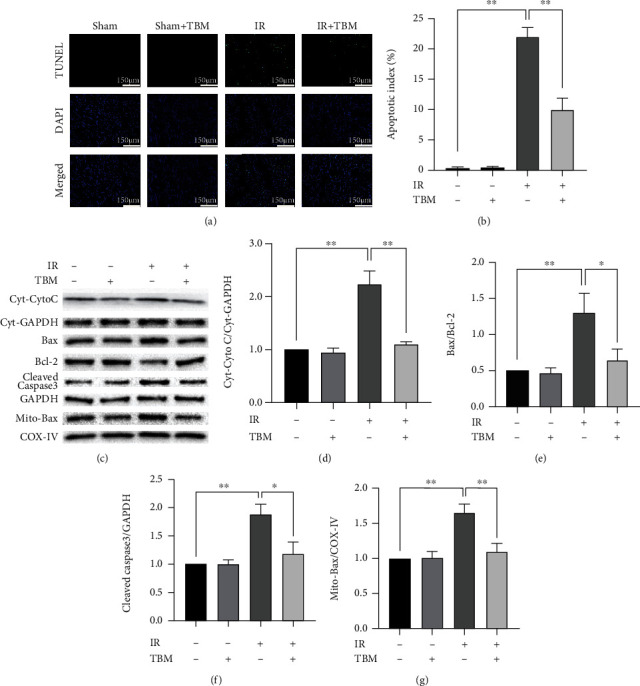
Effect of TBM on cardiac apoptosis and apoptotic signaling pathway in IR. TBM (4 mg/kg) was given at 15 min after ischemia via intraperitoneal injection. After reperfusion, cardiac tissues were detected using TUNEL assay. Total proteins, cytoplasmic proteins, and mitochondrial proteins were extracted and expression of Cyt-Cyto C, Cyt-GAPDH, Bax, Bcl-2, Cleaved Caspase-3, GAPDH, Mito-Bax, and COX-IV was measured using western blotting. (a) Representative images of TUNEL assay. Apoptotic cardiomyocytes were labeled using TUNEL staining, and DAPI was used to detect nuclei. Scar bar: 150 *μ*m. (b) Apoptotic index's statistical analysis. Apoptotic index = (TUNEL‐positive cells/DAPI‐positive cells) × 100%. (c) Representative images of western blotting. (d) Cyt-Cyto C/Cyt-GAPDH's statistical analysis. (e) Bax/Bcl-2's statistical analysis. (f) Cleaved Caspase-3/GAPDH's statistical analysis. (g) Mito-Bax/COX-IV's statistical analysis. All numerical data are expressed as means ± SD, for apoptotic index *n* = 6; for western blotting, *n* = 4. ^∗^*p* < 0.05; ^∗∗^*p* < 0.01.

Reperfusion induces a burst of ROS, and ROS acts on mitochondria, destroys integrity of mitochondria, and releases cytochrome C into the cytoplasm. Cytoplasmic cytochrome C (Cyt-Cyto C) causes apoptosis of cardiac myocytes. As shown in Figure 3, IR induced apoptosis and increased expression of apoptotic proteins, while all these alterations were significantly reversed by TBM. As SIRT3 can inhibit apoptosis by preventing translocation of Bax and TBM induced expression of SIRT3, we supposed that TBM could prevent translocation of Bax. Indeed, IR-increased mitochondrial-located Bax level was significantly inhibited by TBM (Figures 3(c) and 3(g)). These results indicated that TBM reduced IR-caused cardiac apoptosis *in vivo*.

### 3.4. 3-TYP Pretreatment Abolishes TBM's Cardioprotective Effects *In Vivo*

3-TYP is a specific SIRT3 inhibitor. We utilized it to test SIRT3's function in TBM's protective effects. First, 3-TYP's effects on the hearts of sham-operated mice were examined. As shown in Figure S3, LVEF, LVFS, infarct size, plasma LDH levels, oxidative stress, and apoptosis were not influenced by 3-TYP. Furthermore, 3-TYP had little effect on expression of SIRT3, NOX2, Nrf2, NQO1, Bax, Bcl2, Cleaved Caspase-3, Cyt-Cyto C, and Mito-Bax. But 3-TYP decreased SIRT3's activity significantly (Figure S4).

**Figure 4:**
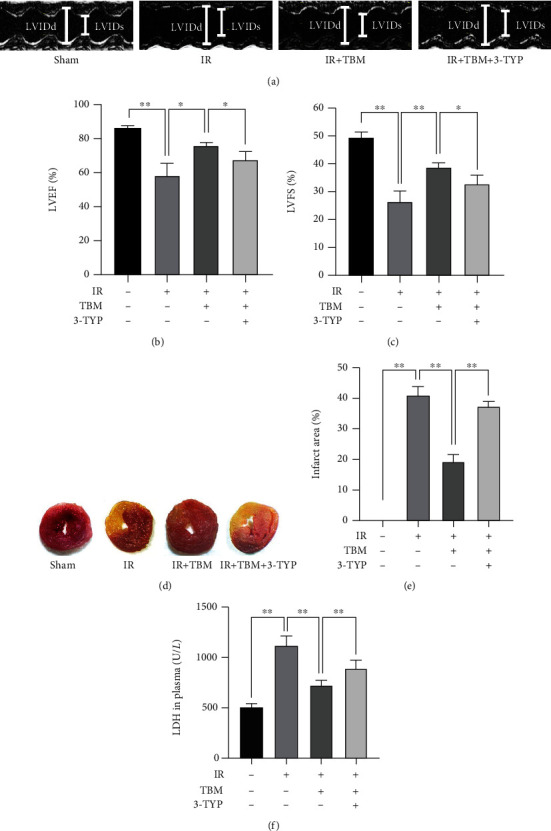
3-TYP pretreatment abolished TBM's cardioprotective effects *in vivo*. Before IR operation, 3-TYP (50 mg/kg) was given per 2 days for a total of 3 times. TBM (4 mg/kg) was given at 15 min after ischemia via intraperitoneal injection. At 24 h postreperfusion, cardiac function, infarct size, and plasma LDH level were determined as described. (a) Representative images of M-mode echocardiography. (b) LVEF's statistical analysis. (c) Statistical analysis of LVFS. (d) Representative images of cardiac sections stained by TTC. Noninfarcted region is red, and the infarcted region is white. (e) Statistical analysis of myocardial infarct size. Infarct area (%) = infarcted region/LV area∗100%. (f) Statistical analysis of plasma LDH level. Data are expressed as means ± SD, *n* = 6. ^∗^*p* < 0.05; ^∗∗^*p* < 0.01.

Then, the influence of 3-TYP on TBM's effects in IR mice was examined. As Figure 4 showed, 3-TYP attenuated TBM's cardioprotective effects, because TBM-increased LVEF and LVFS were attenuated by 3-TYP. Also, compared with the IR+TBM group, mice in the IR+TBM+3-TYP group had increased infarct size and LDH levels. These results indicated that TBM protected the heart through SIRT3.

### 3.5. 3-TYP Pretreatment Abolishes TBM's Antioxidative Effects in IR

**Figure 5:**
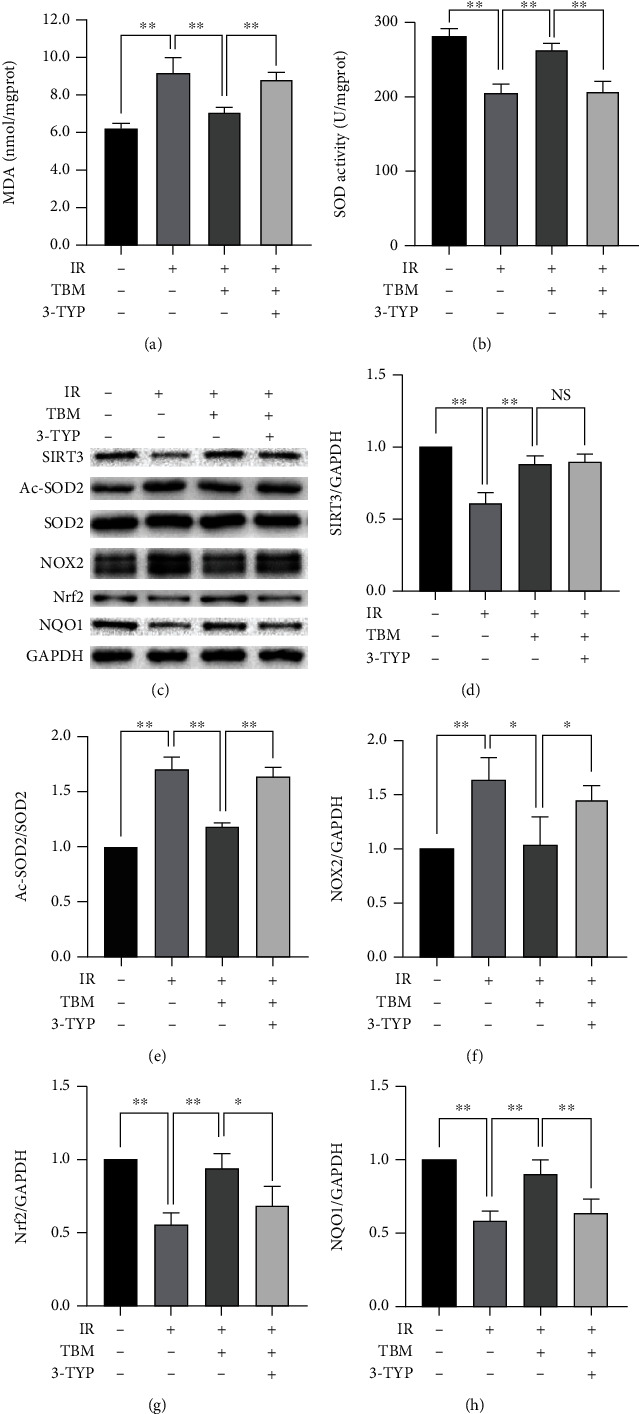
3-TYP pretreatment abolished TBM's antioxidative effects. Before IR operation, 3-TYP (50 mg/kg) was given per 2 days for a total of 3 times. TBM (4 mg/kg) was given at 15 min after ischemia via intraperitoneal injection. At 3 h postreperfusion, MDA content and SOD activity in ischemic myocardial tissue were measured using related kits. Target proteins were examined by western blotting. (a) Statistical analysis of MDA. (b) Statistical analysis of SOD activity. (c) Representative western blotting results. (d) SIRT3/GAPDH's statistical analysis. (e) Ac-SOD2/SOD2's statistical analysis. (f) NOX2/GAPDH's statistical analysis. (g) Nrf2/GAPDH's statistical analysis. (h) NQO1/GAPDH's statistical analysis. All numerical data are expressed as means ± SD, for MDA and SOD activity, *n* = 6; for western blotting, *n* = 4. ^∗^*p* < 0.05; ^∗∗^*p* < 0.01; NS: not significant.

As shown in Figure 5, TBM-induced decrease of MDA was attenuated by 3-TYP. 3-TYP also abolished TBM-increased SOD activity largely. Accordingly, increased antioxidative capacity (increased SIRT3/GAPDH, Nrf2/GAPDH, and NQO1/GAPDH; decreased Ac-SOD2/SOD2 and NOX2/GAPDH) brought by TBM was attenuated by 3-TYP. These results indicated that TBM reduced IR-caused cardiac oxidative stress by activating SIRT3.

### 3.6. 3-TYP Pretreatment Abolishes TBM's Antiapoptotic Effects following IR

**Figure 6:**
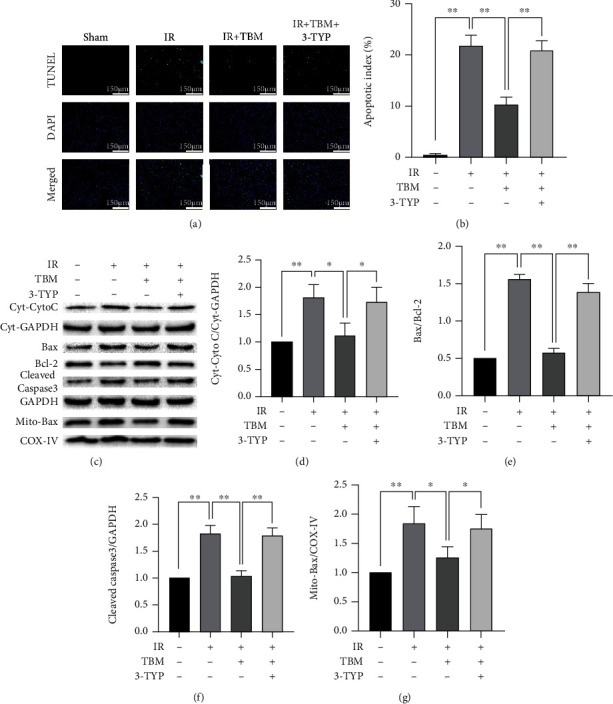
3-TYP pretreatment abolished TBM's antiapoptotic effects. Before IR operation, 3-TYP (50 mg/kg) was given per 2 days for a total of 3 times. TBM (4 mg/kg) was given at 15 min after ischemia via intraperitoneal injection. After reperfusion, cardiac tissues were detected using TUNEL staining. Total proteins, cytoplasmic proteins, and mitochondrial proteins were extracted and expression of Cyt-Cyto C, Cyt-GAPDH, Bax, Bcl-2, Cleaved Caspase-3, GAPDH, Mito-Bax, and COX-IV was measured using western blotting. (a) Representative images of TUNEL assay. Apoptotic cardiomyocytes were labeled using TUNEL staining, and DAPI was used to detect nuclei. Scar bar: 150 *μ*m. (b) Apoptotic index's statistical analysis. Apoptotic index = (TUNEL‐positive cells/DAPI‐positive cells) × 100%. (c) Representative images of western blotting. (d) Cyt-Cyto C/Cyt-GAPDH's statistical analysis. (e) Bax/Bcl-2's statistical analysis. (f) Cleaved Caspase-3/GAPDH's statistical analysis. (g) Mito-Bax/COX-IV's statistical analysis. All numerical data are expressed as means ± SD, for apoptotic index *n* = 6; for western blotting, *n* = 4. ^∗^*p* < 0.05; ^∗∗^*p* < 0.01.

As shown in Figures 6(a) and 6(b), TBM-induced decrease of apoptosis was largely abolished by 3-TYP. Consistently, TBM-induced reductions of proapoptotic proteins were all reversed by 3-TYP (Figures 6(c)–6(g)). These results indicated that TBM reduced IR-caused cardiac apoptosis by activating SIRT3.

### 3.7. TBM's Effects on SIR-Injured H9c2 Cells' Cell Viability, Oxidative Stress, and Apoptosis

**Figure 7:**
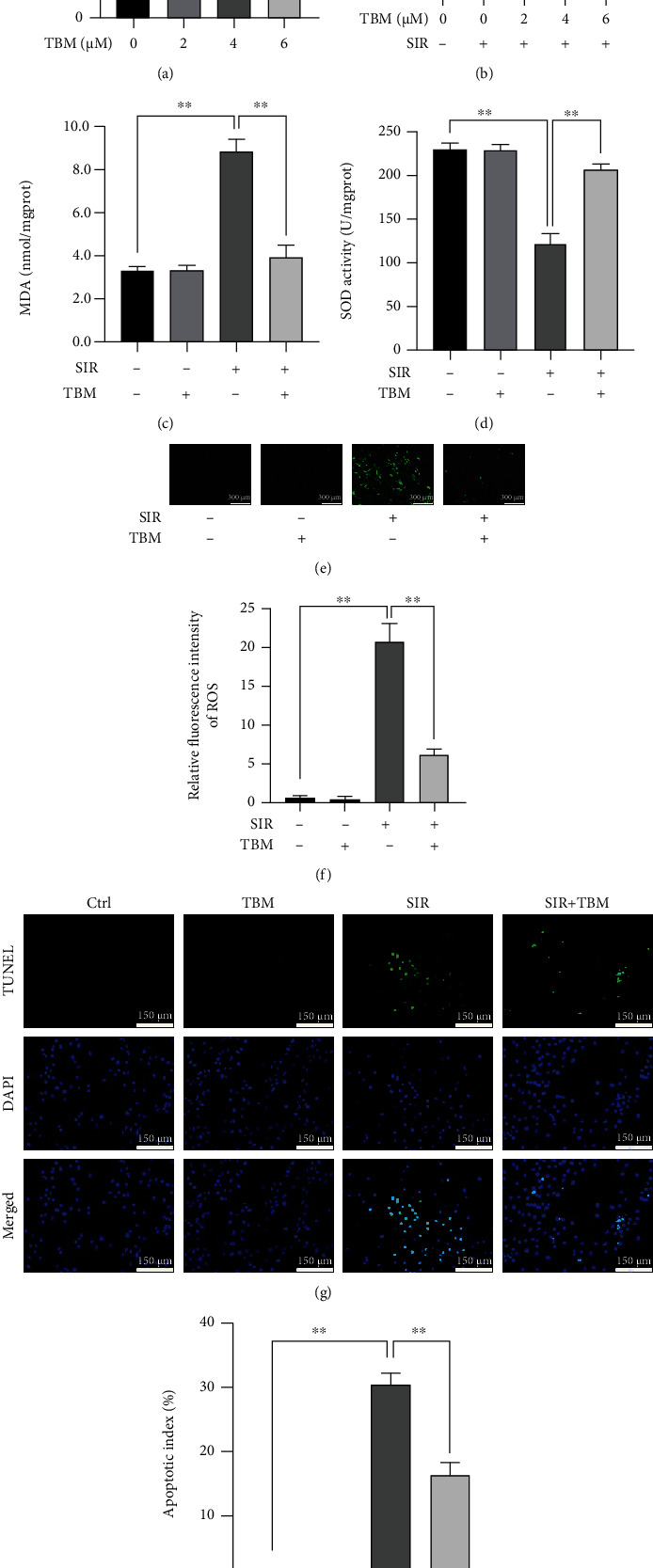
TBM treatment effects on SIR-injured H9c2's cell viability, oxidative stress, and apoptotic index. H9c2 cells were cultivated in DMEM containing 10% FBS. Before experiments, cells were starved in serum-free DMEM for 4 h. To simulate ischemia reperfusion, H9c2 cells were cultivated using ischemic DMEM and low-oxygen incubator (5% CO_2_, 1% O_2_, and 94% N_2_) for 2 h and reperfusion was initiated by replacing the media with complete DMEM and culturing cells under normal air conditions (95% air, 5% CO_2_). Cell viability and apoptotic index were measured at 24 h after reperfusion, while oxidative stress was measured at 4 h after reperfusion. (a) TBM treatment (2-6 *μ*M) had no effect on H9c2's viability under normal condition. (b) TBM treatment (2-6 *μ*M) increased SIR-injured H9c2's cell viability. (c) Intracellular MDA's statistical analysis. (d) Intracellular SOD activity's statistical analysis. (e) Typical ROS staining images. Scar bar: 300 *μ*m. (f) Statistical analysis of ROS's relative fluorescence intensity. (g) Representative images of TUNEL assay. Apoptotic H9c2 was detected using TUNEL staining, and DAPI was used to detect nuclei. Scar bar: 150 *μ*m. (h) Apoptotic index's statistical analysis. Apoptotic index = (TUNEL‐positive cells/DAPI‐positive cells) × 100%. All numerical data are expressed as means ± SD, *n* = 4. ^∗∗^*p* < 0.01.

To illuminate the underlying molecular mechanism, we performed *in vitro* studies using H9c2. First, TBM's effects on normal H9c2 cells' cell viability were examined. As shown in Figure 7(a), TBM had no significant effect on viability of normal H9c2 cells. Then, we treated SIR-injured H9c2 cells with TBM at different concentrations and found that TBM increased cell viability significantly (Figure 7(b)). As 4 *μ*M TBM promoted cell viability most effectively, a dose of 4 *μ*M TBM was used in further mechanism investigations.

**Figure 8:**
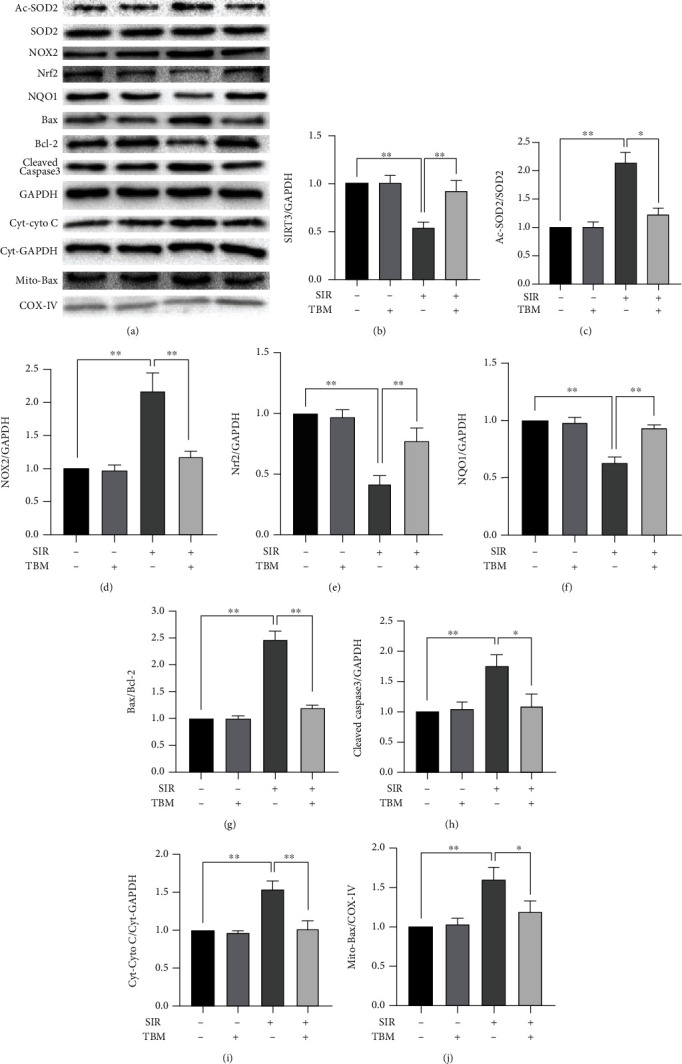
TBM's effects on redox and apoptotic signaling pathway in SIR-injured H9c2 cells. H9c2 were cultivated and treated as Figure 7. At 4 h postreperfusion, cells were harvested. Western blotting was used to detect total proteins, cytoplasmic proteins, and mitochondrial proteins. (a) Representative blots. (b) SIRT3/GAPDH's statistical analysis. (c) Ac-SOD2/SOD2's statistical analysis. (d) NOX2/GAPDH's statistical analysis. (e) Nrf2/GAPDH's statistical analysis. (f) NQO1/GAPDH's statistical analysis. (g) Bax/Bcl-2's statistical analysis. (h) Cleaved Caspase-3/GAPDH's statistical analysis. (i) Statistical analysis of Cyt-Cyto C/Cyt-GAPDH. (j) Statistical analysis of Mito-Bax/COX-IV. All numerical data are expressed as means ± SD, *n* = 4. ^∗^*p* < 0.05; ^∗∗^*p* < 0.01.

Then, we examined TBM's effects on oxidative stress and apoptosis in SIR-treated H9c2 cells.  Figure 7(c) shows TBM's effects on MDA; we can see that TBM inhibited MDA in SIR-injured H9c2 cells. At the same time, TBM increased SOD activity in SIR-injured H9c2 cells (Figure 7(d)). Also, detection of ROS using fluorescent staining demonstrated that TBM could reduce ROS in SIR-injured H9c2 cells (Figures 7(e) and 7(f)). Further, we detected expression of proteins related with redox balance. As shown in Figures 8(a)–8(f), SIR-decreased expression of SIRT3, Nrf2, and NQO1 and increased expression of oxidative protein NOX2 brought by SIR were reversed by TBM. Figures 7(g) and 7(h) show TBM's effects on apoptosis in SIR-treated H9c2 cells. Compared with that in the SIR group, the number of apoptotic cells in the SIR+TBM group is much less. Also, TBM's effects on expression of apoptotic proteins were detected. As Figures 8(a) and 8(g)–8(j) showed, induced Bax, Cleaved Caspase-3, Cyt-Cyto C, and Mito-Bax in SIR-injured H9c2 cells were all reversed by TBM. All results shown in Figures 7 demonstrated that TBM could improve SIR-treated H9c2 cells' cell viability by inhibiting oxidative stress and apoptosis. All these results were consistent with our *in vivo* findings.

### 3.8. SIRT3 Is an Essential Factor in TBM's Protective Effects in SIR-Injured H9c2 Cells

**Figure 9:**
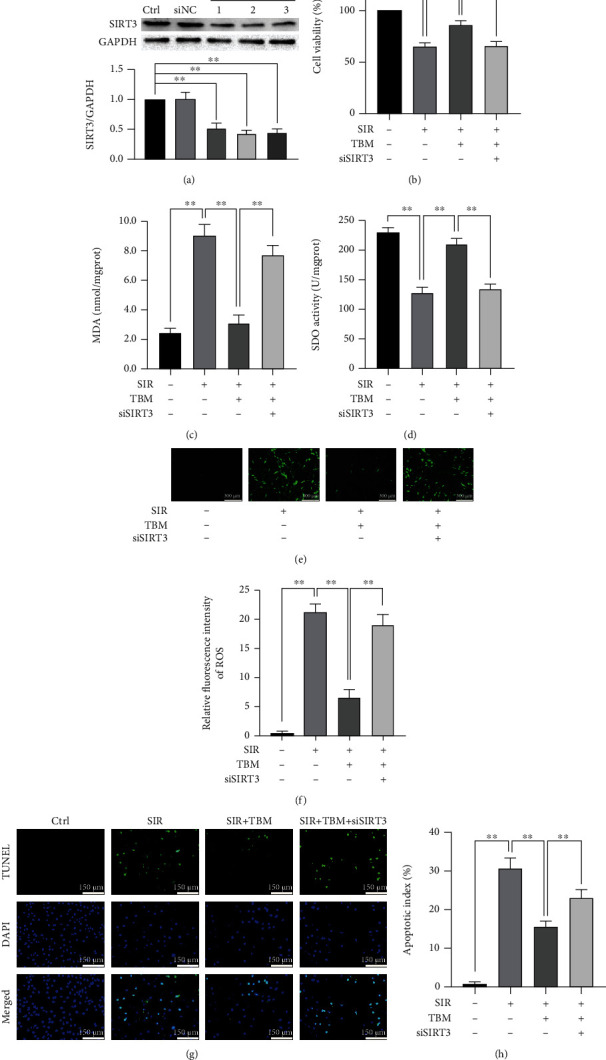
In SIR-injured H9c2 cells, SIRT3 siRNA abolished TBM's cytoprotective effects. H9c2 cultured in DMEM containing 10% FBS were transfected with siSIRT3. Then, transfected cells were treated with TBM or SIR as described. (a) Confirmation of siSIRT3's efficiency using western blotting. (b) CCK8 kit examined cell viability. (c) Intracellular MDA's statistical analysis. (d) Intracellular SOD activity's statistical analysis. (e) Typical ROS staining images. Scar bar: 300 *μ*m. (f) Statistical analysis of relative fluorescence intensity of ROS. (g) Representative images of TUNEL assay. Apoptotic H9c2 was detected using TUNEL assay, and DAPI was used to detect nuclei. Scar bar: 150 *μ*m. (h) Apoptotic index's statistical analysis. Apoptotic index = (TUNEL‐positive cells/DAPI‐positive cells) × 100%. All numerical data are expressed as means ± SD, *n* = 4. ^∗∗^*p* < 0.01.

**Figure 10:**
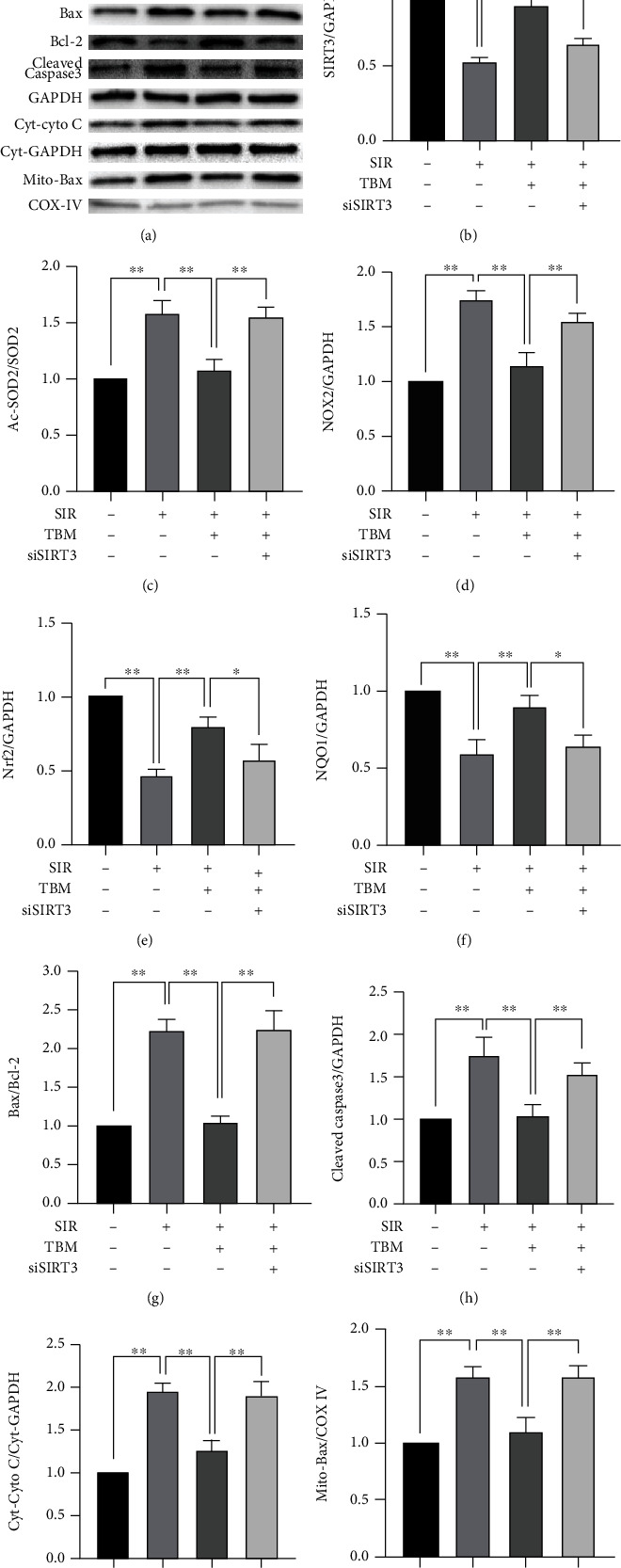
In SIR-injured H9c2 cells, SIRT3 siRNA abolished TBM's effects on redox and apoptotic signaling pathway. H9c2 cultured in DMEM containing 10% FBS were transfected with siSIRT3. Then, transfected cells were treated with TBM or SIR as described. (a) Representative blots. (b) SIRT3/GAPDH's statistical analysis. (c) Ac-SOD2/SOD2's statistical analysis. (d) NOX2/GAPDH's statistical analysis. (e) Nrf2/GAPDH's statistical analysis. (f) NQO1/GAPDH's statistical analysis. (g) Bax/Bcl-2's statistical analysis. (h) Cleaved Caspase-3/GAPDH's statistical analysis. (i) Statistical analysis of Cyt-Cyto C/Cyt-GAPDH. (j) Statistical analysis of Mito-Bax/COX-IV. All numerical data are expressed as means ± SD, *n* = 4. ^∗^*p* < 0.05; ^∗∗^*p* < 0.01.

To figure out SIRT3's role in TBM's protective effects in SIR-injured H9c2 cells, SIRT3 was knocked down by siRNA. First, three siRNAs were designed and synthesized, and their efficiency and specificity were confirmed by western blotting. As Figure 9(a) showed, SIRT3 could be knocked down by all three siRNAs efficiently and specifically. Then, the role of SIRT3 in TBM's protective effects was examined. As Figures 9(b)–9(h) showed, TBM's prosurvival, antioxidative, and antiapoptotic effects were all reduced by siSIRT3. Additionally, siSIRT3 abolished TBM's inhibitive effects on NOX2 expression and Ac-SOD2/SOD2 ratio (Figures 10(a)–10(c)). Also, siSIRT3 attenuated enhanced expression of antioxidative proteins Nrf2 and NQO1 brought by TBM (Figures 10(e) and 10(f)).

Also, siSIRT3 abolished TBM's antiapoptotic effects largely. As shown in Figures 10(a) and 10(g)–10(j), Bax/Bcl-2, Cleaved Caspase-3/GAPDH, Cyt-Cyto C/Cyt-GAPDH, and Mito-Bax/COX IV were increased significantly by siSIRT3. These results indicated that SIRT3 played essential roles in TBM's protective effects against IR-induced cardiac dysfunction.

## 4. Discussion

In this study, we examined TBM's effects on MIRI both *in vivo* and *in vitro*. *In vivo*, TBM administration improved postischemic cardiac function, decreased myocardial infarct size, reduced plasma LDH levels, reduced oxidative stress, and reduced apoptotic cardiomyocyte number. *In vitro*, TBM improved cell viability, decreased oxidative stress, and reduced apoptotic H9c2 number. TBM's protective effects are mediated, at least partially, by activating SIRT3, as both 3-TYP, a specific SIRT3 inhibitor, and siSIRT3 abolished TBM's protective effects remarkably.

It is well accepted that oxidative stress plays a central role in MIRI. Increased ROS production and decreased ROS scavenging ability lead to oxidative stress [40]. In this study, TBM protected against MIRI by reducing oxidative stress. On the one hand, TBM decreased ROS production by inhibiting NOX2. On the other hand, TBM increased antioxidant factors such as Nrf2 and NQO1. As far as we know, this is the first report that TBM is of antioxidative activity.

Among 7 sirtuins, SIRT3 has gained more attention due to its mitochondrial location and involvement in extending human lifespan [41]. In MIR rats, SIRT3 is downregulated [17]. Upregulation of SIRT3 ameliorates MIRI through inhibiting mPTP opening by deacetylating cyclophilin D [21]. Also, SIRT3 deficiency exacerbates MIRI [18]. All these studies demonstrated that SIRT3 played vital role in MIRI. SIRT3 induces Nrf2 which is responsible for transcription of important antioxidant factors. These antioxidant factors fight against oxidative stress [42–44]. SIRT3 elevates SOD2's activity by deacetylation [45]. SIRT3 also inhibits apoptosis. First, overexpression of SIRT3 increases the ratio of Bcl-2/Bax [46]. Second, SIRT3 prevents translocation of Bax to mitochondria by deacetylating Ku70 [24]. Third, SIRT3 acetyls cyclophilin D and prevents release of mitochondrial cytochrome C into the cytoplasm [21]. In this study, we found that TBM administration ameliorated MIRI by reducing oxidative stress and apoptosis and all these protective effects were abolished by SIRT3 inhibition. All these results suggested that Sirt3-mediated antioxidative and antiapoptotic mechanisms contributed to TBM's protective effects.

Previous studies on TBM focused on its antitumor activity [27]. And most of these reports demonstrated that TBM induced oxidative stress and apoptosis [47, 48]. Our results seem to contradict with previous reports. These contradictions can be explained by SIRT3's diversified functions. On the one hand, SIRT3 can inhibit apoptosis. SIRT3 interacts with OGG1, repairs mitochondrial DNA, and protects from apoptotic cell death under oxidative stress [49]. In cardiomyocytes, SIRT3 protects cells from stress-mediated apoptosis by deacetylation of Ku70 [24]. SIRT3 rescues p53-induced growth arrest in human bladder tumor-derived EJ-p53 cells [50]. SIRT3 promotes cancer cell survival by inhibiting apoptosis [51, 52]. On the other hand, SIRT3 can promote apoptosis. Under basal conditions, SIRT3 promotes apoptosis by regulating cell survival pathways [53]. SIRT3 promotes cancer cell apoptosis through destabilizing HIF1*α* [54]. SIRT3 promotes hepatocellular carcinoma apoptosis by reducing Mdm2-mediated p53 degradation [55]. In different cell types or under different conditions, SIRT3 can inhibit or promote apoptosis, because that TBM work through SIRT3. It is reasonable that TBM promotes oxidative stress and apoptosis in cancer cells while TBM inhibits oxidative stress and apoptosis in cardiomyocytes.

Besides TBM, trans sodium crocetinate, total salvianolic acid, dihydromyricetin, 3-bromo-4,5-dihydroxybenzaldehyde, exendin-4, and melatonin have been reported to protect against MIRI via SIRT3 [56–62]. All these drugs induce expression of SIRT3, but their mechanisms are different. Total salvianolic acid, exendin-4, and melatonin induce expression of both SIRT1 and SIRT3. In our study, we found that IR decreased expression of SIRT1, but TBM could not reverse expression of SIRT1 (Figure S5). Recently, TBM has been reported to antagonize Yoda1-evoked Piezo1 channel activation [63]. Also, Piezo1 is proposed to be a vital factor that leads to cerebral ischemia-reperfusion injury [64]. We speculated that TBM induced expression of SIRT3 by binding to Piezo1. This hypothesis will be tested in the future.

With the development of stent interventional therapy, new antiplatelet agents, and antithrombotic agents, myocardial reperfusion therapy has been greatly optimized. But there is still no effective treatment to prevent MIRI. Cyclosporine A, an inhibitor of cyclophilin D (a protein controlling mPTP's opening), alleviates MIRI in cardiac IR animal models [65, 66]. However, a large multicentric clinical trial investigating cyclosporine A's efficacy reveals no protective effects of the drug on clinical outcomes in MI patients [67]. Metoprolol, a *β*1 receptor blocker, reduces MIRI. But it requires long transporting times [68]. In the present study, our results demonstrate that TBM alleviates MIRI by reducing oxidative stress and apoptosis in a short term. Previously, we have reported that TBM promoted angiogenesis via the eNOS-VEGF signaling pathway [39]. In the long term, TBM's angiogenic effect might promote AMI patients' rehabilitation. All our results indicate that TBM might be a potent new drug against MIRI.

There are four limits that need to be addressed in our study. First, to confirm SIRT3's function, more intensive methods such as genetic knockout mice should be used. Second, to reproduce physiological state, primary cardiomyocytes should be used in *in vitro* experiments. Third, we did not figure out TBM's direct target. This will be studied in the future. Fourth, SIRT3 has a long isoform (~44 kDa) and a short isoform (~28 kDa). In mitochondria, the short isoform is more likely to exhibit deacetylase activity and it is the main one [69]. So, we examined SIRT3's short isoform in this study. However, it has been reported that discrepancy of the two SIRT3 isoforms existed in a cardiac hypertrophy model [70]. We will pay attention to these two isoforms in future studies.

In a word, for the first time, our data show that TBM administration inhibits IR-induced cardiac dysfunction, oxidative stress, and apoptosis through SIRT3. Our understanding of TBM's effects has been broadened, and this study provided molecular evidence to develop TBM as a therapeutic intervention of MIRI in AMI patients.

## Data Availability

All authors confirm that the data supporting the findings of the study are provided within the manuscript and the supplementary file.

## Supplementary Materials

Supplementary Materials Figure S1: *in vivo* experimental protocol. Figure S2: *in vitro* experimental protocol. Figure S3: TBM and 3-TYP's effects on cardiac function, myocardial infarct size, oxidative stress, and apoptosis in sham group mice. TBM (4 mg/kg) or 3-TYP (50 mg/kg) was intraperitoneally injected into sham operation mice, and cardiac function, myocardial infarct size, oxidative stress, and apoptosis were determined after sham operation. (A) Representative M-mode images of echocardiography for each group. (B) Statistical analysis of LVEF. Data are expressed as means ± SD, *n* = 6. (C) Statistical analysis of LVFS. Data are expressed as means ± SD, *n* = 6. (D) Representative images of heart sections stained by TTC. (E) Statistical analysis of myocardial infarct size expressed as a percentage of infarcted region/LV area. (F) LDH levels in plasma. Data are expressed as means ± SD, *n* = 6. (G) MDA levels in myocardial tissue. Data are expressed as means ± SD, *n* = 6. (H) Activity of SOD in myocardial tissue. Data are expressed as means ± SD, *n* = 6. ^∗∗^*p* < 0.01. (I) Representative images of TUNEL staining. Scar bar: 150 *μ*m. (J) Statistical analysis of apoptotic index. Data are expressed as means ± SD, *n* = 6. Figure S4: TBM and 3-TYP's effects on the SIRT3/SOD2, oxidative stress, and apoptotic signaling pathways in sham group mice. TBM (4 mg/kg) or 3-TYP (50 mg/kg) was intraperitoneally injected into sham operation mice, and myocardial tissue was examined by western blotting. (A) Representative western blotting results. (B) Statistical analysis of SIRT3/GAPDH. Data are expressed as means ± SD, *n* = 4. (C) Statistical analysis of Ac-SOD2/SOD2. Data are expressed as means ± SD, *n* = 4. ^∗∗^*p* < 0.01. (D) Statistical analysis of NOX2/GAPDH. Data are expressed as means ± SD, *n* = 4. (E) Statistical analysis of Nrf2/GAPDH. Data are expressed as means ± SD, *n* = 4. (F) Statistical analysis of NQO1/GAPDH. Data are expressed as means ± SD, *n* = 4. (G) Statistical analysis of Bax/Bcl-2. Data are expressed as means ± SD, *n* = 4. (H) Statistical analysis of Cleaved Caspase-3/GAPDH. Data are expressed as means ± SD, *n* = 4. (I) Statistical analysis of Cyt-Cyto C/Cyt-GAPDH. Data are expressed as means ± SD, *n* = 4. (J) Statistical analysis of Mito-Bax/COX-IV. Data are expressed as means ± SD, *n* = 4. Figure S5: TBM did not reverse expression of SIRT1. TBM (4 mg/kg) was given at 15 min after ischemia via intraperitoneal injection. After reperfusion, cardiac tissues were examined by western blotting. (A) Representative western blotting results of SIRT1. (B) Statistical analysis of SIRT1/GAPDH in each group. Data are expressed as means ± SD, *n* = 4. ^∗∗^*p* < 0.01; NS: not significant. H9c2 were cultured and treated as described in Materials and Methods. Then, cells were collected for detection using western blotting. (C) Representative western blotting results of SIRT1. (D) Statistical analysis of SIRT1/GAPDH in each group. Data are expressed as means ± SD, *n* = 4. ^∗∗^*p* < 0.01; NS: not significant.
