# Supplementary material for: Tubeimoside I Ameliorates Myocardial Ischemia-Reperfusion Injury through SIRT3-Dependent Regulation of Oxidative Stress and Apoptosis
Source: Oxid Med Cell Longev. 2021 Nov 9;2021:5577019. doi: 10.1155/2021/5577019 (PMC8595016; doi:10.1155/2021/5577019)
Supplement: Supplementary Materials — Figure S1: in vivo experimental protocol. Figure S2: in vitro experimental protocol. Figure S3: TBM and 3-TYP's effects on cardiac function, myocardial infarct size, oxidative stress, and apoptosis in sham group mice. TBM (4 mg/kg) or 3-TYP (50 mg/kg) was intraperitoneally injected into sham operation mice, and cardiac function, myocardial infarct size, oxidative stress, and apoptosis were determined after sham operation. (A) Representative M-mode images of echocardiography for each group. (B) Statistical analysis of LVEF. Data are expressed as means ± SD, n = 6. (C) Statistical analysis of LVFS. Data are expressed as means ± SD, n = 6. (D) Representative images of heart sections stained by TTC. (E) Statistical analysis of myocardial infarct size expressed as a percentage of infarcted region/LV area. (F) LDH levels in plasma. Data are expressed as means ± SD, n = 6. (G) MDA levels in myocardial tissue. Data are expressed as means ± SD, n = 6. (H) Activity of SOD in myocardial tissue. Data are expressed as means ± SD, n = 6. ∗∗p < 0.01. (I) Representative images of TUNEL staining. Scar bar: 150 μm. (J) Statistical analysis of apoptotic index. Data are expressed as means ± SD, n = 6. Figure S4: TBM and 3-TYP's effects on the SIRT3/SOD2, oxidative stress, and apoptotic signaling pathways in sham group mice. TBM (4 mg/kg) or 3-TYP (50 mg/kg) was intraperitoneally injected into sham operation mice, and myocardial tissue was examined by western blotting. (A) Representative western blotting results. (B) Statistical analysis of SIRT3/GAPDH. Data are expressed as means ± SD, n = 4. (C) Statistical analysis of Ac-SOD2/SOD2. Data are expressed as means ± SD, n = 4. ∗∗p < 0.01. (D) Statistical analysis of NOX2/GAPDH. Data are expressed as means ± SD, n = 4. (E) Statistical analysis of Nrf2/GAPDH. Data are expressed as means ± SD, n = 4. (F) Statistical analysis of NQO1/GAPDH. Data are expressed as means ± SD, n = 4. (G) Statistical analysis of Bax/Bcl-2. Data are expressed as m [file 5577019.f1.zip › Fig S2.docx]

Ctrl

TBM

SIR

SIR+TBM

SIR+TBM+siSIRT3

siSIRT3

Time (hours)

-1

0

2

24

time2

time3

time4

-4

time1

DMSO

DMSO

DMSO

TBM (4 μM)

time6

Note:

1. At time1, cells were starved in DMEM without serum;

2. At time2, DMSO or TBM were added into DMEM without serum;

3. At time3, simulated ischemia start. Cells in Ctrl, TBM and siSIRT3 group were cultivated in complete DMEM (containing 4.5 g glucose and 10% FBS) supplemented with or without TBM and they were cultured under normal air condition (95% air and 5% CO_2_). Cells in SIR, SIR+TBM and SIR+TBM+siSIRT3 group were cultivated in ischemic DMEM supplemented with or without TBM and they were cultured in low-oxygen incubator (1% O_2_, 94% N_2_ and 5% CO_2_). Ischemic DMEM is DMEM without glucose and serum and dissolved oxygen in the culture media was expelled by filling nitrogen;

4. At time4, all cells were cultured in complete DMEM (containing 4.5 g glucose and 10% FBS) supplemented with or without TBM. And all cells were cultivated under normal air condition (95% air and 5% CO_2_);

5. At time5, cells were collected for western blotting and oxidative stress measurement;

6. At time6, cells were collected for cell viability and apoptotic index detection.

siSIRT3

siSIRT3

TBM (4 μM)

TBM (4 μM)

6

time5

**Figure S2**
