# Supplementary figures and images for: Tubeimoside I Ameliorates Myocardial Ischemia-Reperfusion Injury through SIRT3-Dependent Regulation of Oxidative Stress and Apoptosis
Source: Oxid Med Cell Longev. 2021 Nov 9;2021:5577019. doi: 10.1155/2021/5577019 (PMC8595016; doi:10.1155/2021/5577019)

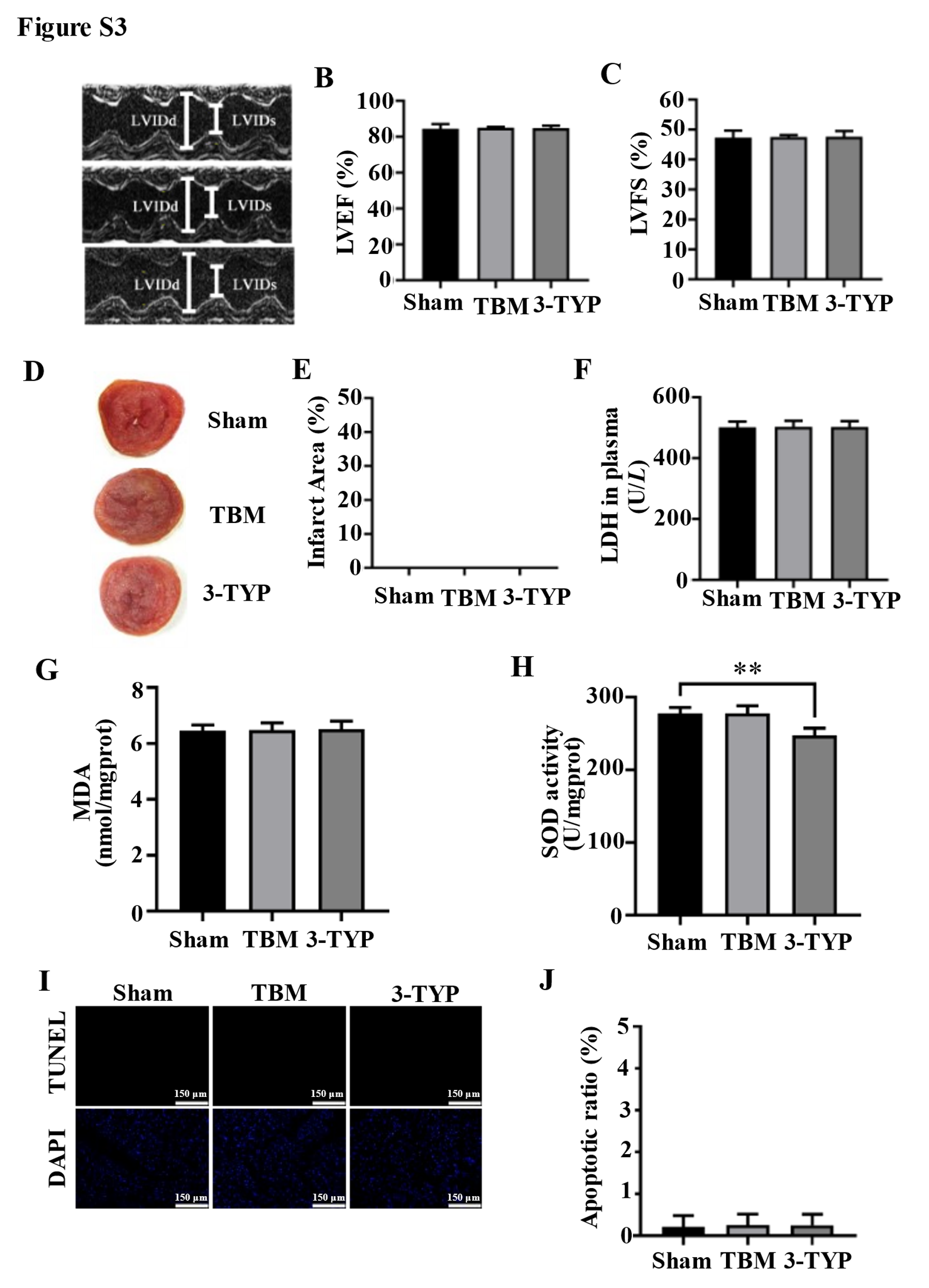

Supplement: Supplementary Materials — Figure S1: in vivo experimental protocol. Figure S2: in vitro experimental protocol. Figure S3: TBM and 3-TYP's effects on cardiac function, myocardial infarct size, oxidative stress, and apoptosis in sham group mice. TBM (4 mg/kg) or 3-TYP (50 mg/kg) was intraperitoneally injected into sham operation mice, and cardiac function, myocardial infarct size, oxidative stress, and apoptosis were determined after sham operation. (A) Representative M-mode images of echocardiography for each group. (B) Statistical analysis of LVEF. Data are expressed as means ± SD, n = 6. (C) Statistical analysis of LVFS. Data are expressed as means ± SD, n = 6. (D) Representative images of heart sections stained by TTC. (E) Statistical analysis of myocardial infarct size expressed as a percentage of infarcted region/LV area. (F) LDH levels in plasma. Data are expressed as means ± SD, n = 6. (G) MDA levels in myocardial tissue. Data are expressed as means ± SD, n = 6. (H) Activity of SOD in myocardial tissue. Data are expressed as means ± SD, n = 6. ∗∗p < 0.01. (I) Representative images of TUNEL staining. Scar bar: 150 μm. (J) Statistical analysis of apoptotic index. Data are expressed as means ± SD, n = 6. Figure S4: TBM and 3-TYP's effects on the SIRT3/SOD2, oxidative stress, and apoptotic signaling pathways in sham group mice. TBM (4 mg/kg) or 3-TYP (50 mg/kg) was intraperitoneally injected into sham operation mice, and myocardial tissue was examined by western blotting. (A) Representative western blotting results. (B) Statistical analysis of SIRT3/GAPDH. Data are expressed as means ± SD, n = 4. (C) Statistical analysis of Ac-SOD2/SOD2. Data are expressed as means ± SD, n = 4. ∗∗p < 0.01. (D) Statistical analysis of NOX2/GAPDH. Data are expressed as means ± SD, n = 4. (E) Statistical analysis of Nrf2/GAPDH. Data are expressed as means ± SD, n = 4. (F) Statistical analysis of NQO1/GAPDH. Data are expressed as means ± SD, n = 4. (G) Statistical analysis of Bax/Bcl-2. Data are expressed as m [file 5577019.f1.zip › Fig S3.docx]

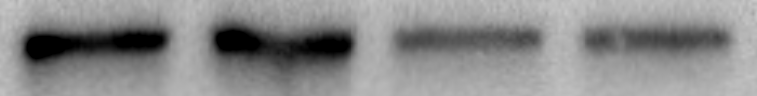

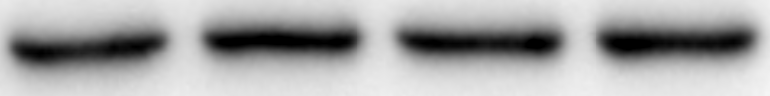

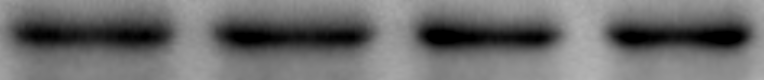

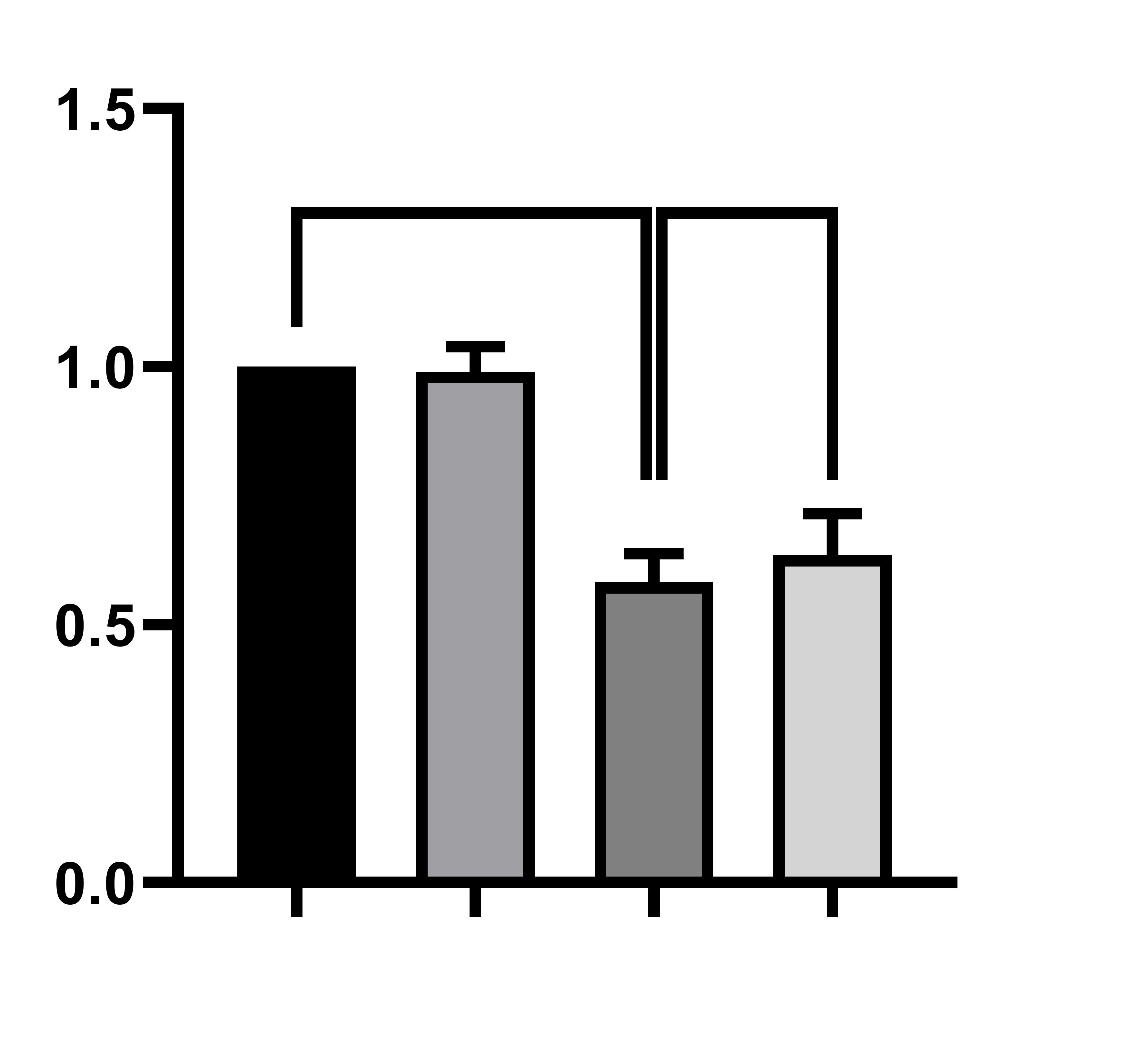

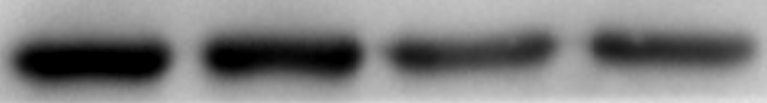


**Figure S5**

**IR**

**TBM**

**−**

**−**

**−**

**−**

**+**

**+**

**+**

**+**

**A**

**SIRT1**

**GAPDH**

**B**

**SIRT1**

**GAPDH**

**TBM**

**IR**

**-**

**-**

**+**

**+**

**-**

**+**

**-**

**+**

**1.0**

**0.5**

**0.0**

**SIRT3/GAPDH**

**1.5**


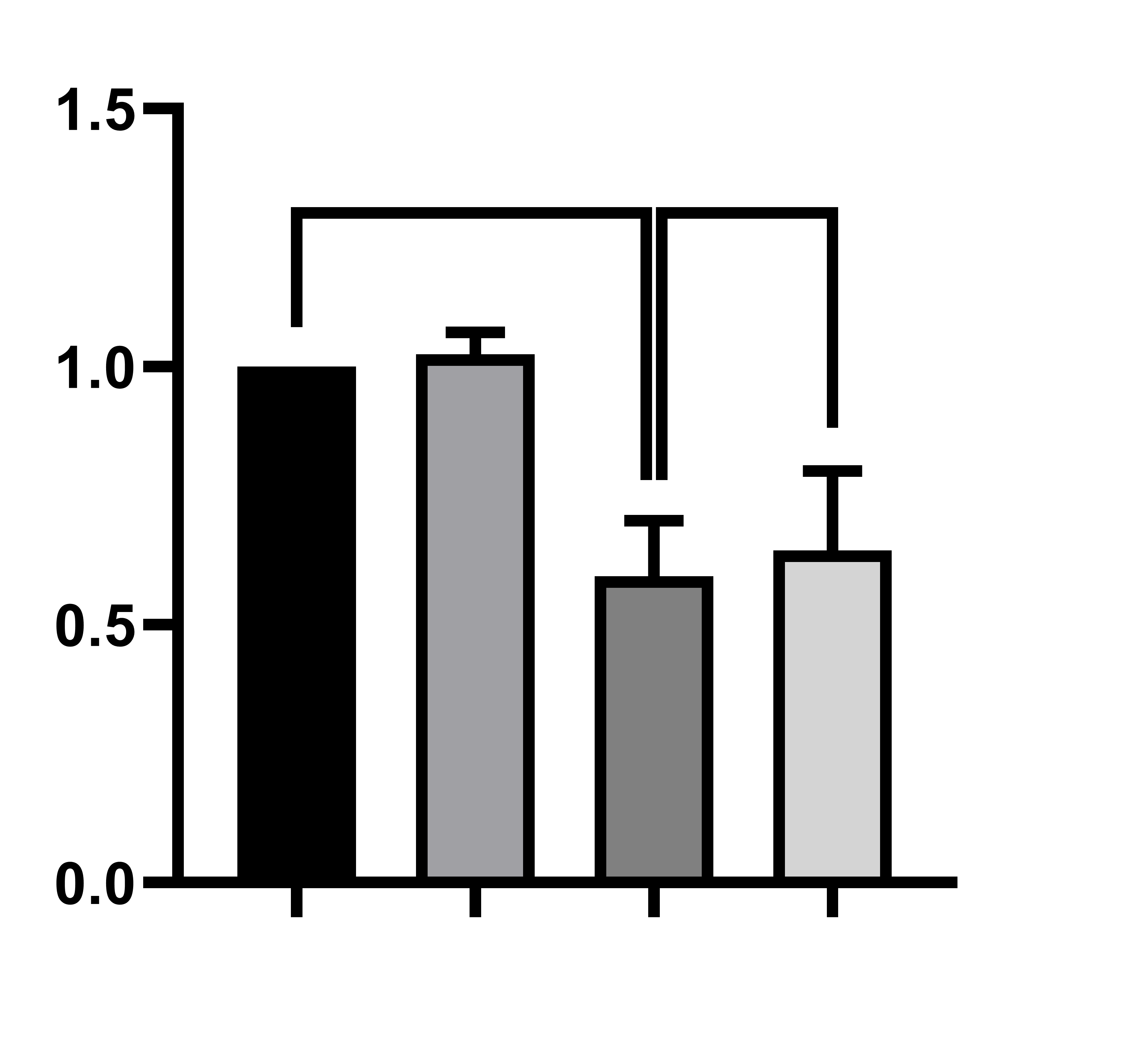


**1.0**

**0.5**

**0.0**

**SIRT1/GAPDH**

**1.5**

**TBM**

**SIR**

**-**

**-**

**+**

**+**

**-**

**+**

**-**

**+**

**C**

**D**

**

**NS**

**NS**

**

**SIR**

**TBM**

**−**

**−**

**−**

**−**

**+**

**+**

**+**

**+**

Supplement: Supplementary Materials — Figure S1: in vivo experimental protocol. Figure S2: in vitro experimental protocol. Figure S3: TBM and 3-TYP's effects on cardiac function, myocardial infarct size, oxidative stress, and apoptosis in sham group mice. TBM (4 mg/kg) or 3-TYP (50 mg/kg) was intraperitoneally injected into sham operation mice, and cardiac function, myocardial infarct size, oxidative stress, and apoptosis were determined after sham operation. (A) Representative M-mode images of echocardiography for each group. (B) Statistical analysis of LVEF. Data are expressed as means ± SD, n = 6. (C) Statistical analysis of LVFS. Data are expressed as means ± SD, n = 6. (D) Representative images of heart sections stained by TTC. (E) Statistical analysis of myocardial infarct size expressed as a percentage of infarcted region/LV area. (F) LDH levels in plasma. Data are expressed as means ± SD, n = 6. (G) MDA levels in myocardial tissue. Data are expressed as means ± SD, n = 6. (H) Activity of SOD in myocardial tissue. Data are expressed as means ± SD, n = 6. ∗∗p < 0.01. (I) Representative images of TUNEL staining. Scar bar: 150 μm. (J) Statistical analysis of apoptotic index. Data are expressed as means ± SD, n = 6. Figure S4: TBM and 3-TYP's effects on the SIRT3/SOD2, oxidative stress, and apoptotic signaling pathways in sham group mice. TBM (4 mg/kg) or 3-TYP (50 mg/kg) was intraperitoneally injected into sham operation mice, and myocardial tissue was examined by western blotting. (A) Representative western blotting results. (B) Statistical analysis of SIRT3/GAPDH. Data are expressed as means ± SD, n = 4. (C) Statistical analysis of Ac-SOD2/SOD2. Data are expressed as means ± SD, n = 4. ∗∗p < 0.01. (D) Statistical analysis of NOX2/GAPDH. Data are expressed as means ± SD, n = 4. (E) Statistical analysis of Nrf2/GAPDH. Data are expressed as means ± SD, n = 4. (F) Statistical analysis of NQO1/GAPDH. Data are expressed as means ± SD, n = 4. (G) Statistical analysis of Bax/Bcl-2. Data are expressed as m [file 5577019.f1.zip › Fig S5.docx]
